# IVA-FL: An information-value-aware federated learning framework for Privacy-Preserving Financial data Risk Management

**DOI:** 10.1371/journal.pone.0344251

**Published:** 2026-03-20

**Authors:** Jinyu Wu, Hanjie Xu, Tianfan Zhang, Ronghu Xu, Haozheng Wu, Zhengxi Huang

**Affiliations:** 1 School of Accounting, Guangzhou Xinhua University, Guangzhou, Guangdong, China; 2 Department of Artificial Intelligence and Data Science, Guangzhou Xinhua University, Guangzhou, Guangdong, China; 3 College of Computer Science, Guangdong Polytechnic Normal University, Guangzhou, Guangdong, China; 4 Department of Education Technology, Qingdao Hengxing University, Qingdao, Shandong, China; Vellore Institute of Technology - Chennai Campus, INDIA

## Abstract

Federated Learning (FL) has emerged as a critical paradigm for enabling financial institutions to collaboratively train models while preserving data privacy. However, a fundamental challenge arises when applying Differential Privacy (DP) to financial risk management tasks characterized by severe class imbalance. The uniform noise injection of standard DP mechanisms disproportionately submerges the critical risk signals from rare events such as fraud or default. To address this, we introduce IVA-FL (Information-Value-Aware Federated Learning), a novel framework that shifts the privacy preservation paradigm from data-agnostic optimization to a value-driven approach. IVA-FL integrates three synergistic mechanisms: 1) an Information Value Scoring (IVS) mechanism that dynamically quantifies the importance of each sample based on real-time training loss; 2) an Adaptive Gradient Processing module that applies tailored clipping and smoothing strategies to rescue high-value gradients; and 3) an Adaptive Noise Injection mechanism that intelligently reallocates the scarce privacy budget to maximize the signal-to-noise ratio of critical risks. Comprehensive experiments on multiple financial datasets demonstrate that IVA-FL significantly outperforms state-of-the-art baselines in both standard metrics (Recall, AUC) and practical risk assessment indicators (KS Statistic, Brier Score), especially under strict privacy constraints. Furthermore, the framework exhibits exceptional robustness against Data Heterogeneity (Non-IID) and superior adaptability in advanced scenarios involving feature heterogeneity and concept drift. Our work presents a robust, high-utility solution for privacy-preserving financial risk management.

## Introduction

In the modern financial system, risk management models driven by artificial intelligence and big data have become the core engine for maintaining financial stability and enhancing service efficiency [1]. Financial institutions have accumulated vast amounts of user data, which are crucial for building accurate models for credit scoring, fraud detection, and credit default prediction [2]. However, due to increasingly stringent regulations and the sensitive nature of commercial competition, this valuable data is often confined within individual institutions, creating the phenomenon known as data silos [3]. This severely limits the potential for cross-institutional collaborative modeling to identify systemic and cross-platform risks. In response to this challenge, Federated Learning (FL) has emerged as a novel distributed machine learning paradigm [4]. It enables multiple parties to collaboratively train a global model by exchanging model updates without sharing their raw data, thereby providing a foundational technical guarantee for data privacy while breaking down data barriers [5]. However, as emphasized in the work of Awosika et al., a peculiar characteristic of the financial risk control domain is its extremely imbalanced data: fraudulent transactions often account for less than 1% of all transaction records [6]. This intrinsic asymmetry implies that the cost of a single False Negative (i.e., failing to detect a fraudulent transaction) can outweigh the cumulative benefits of thousands of True Negatives (i.e., correctly identifying legitimate transactions). This renders traditional accuracy metrics inadequate and places the pursuit of high model Recall in a position of paramount importance [7]. Consequently, a critical and unresolved challenge is how to, within the FL framework, satisfy stringent privacy requirements while ensuring the model possesses high sensitivity and learning capability for these rare yet crucial risk signals [8].

To provide a quantifiable and rigorous privacy guarantee in federated learning, Differential Privacy (DP) has become the recognized gold standard in both academia and industry. By injecting precisely calibrated noise into gradient updates, DP can effectively prevent an adversary from inferring sensitive information about any single user from the shared model parameters [9]. However, when this powerful privacy-preserving mechanism is applied to tasks with highly non-uniform information value, such as financial risk control, it reveals a fundamental inherent contradiction [10]. From an information-theoretic perspective, the standard DP mechanism introduces an isotropic noise field—akin to a uniform “fog”—across the high-dimensional gradient space [11]. While this fog is transparent enough to reveal the “mountains” (gradients from abundant normal samples), it is thick enough to completely obscure the “faint beacons” (gradients from rare fraud samples). When the signal carrying critical information has an energy level far below the average energy of the noise field, the Signal-to-Noise Ratio (SNR) for recovering this signal at the aggregation server deteriorates sharply. This makes the effective learning of these key patterns statistically intractable. The direct consequence is a precipitous decline in the model’s recall metric, which is unacceptable in risk-sensitive commercial applications [12,13].

The academic community has widely recognized the dual challenges of data imbalance and privacy preservation in federated learning and has explored solutions from multiple perspectives [14]. One mainstream line of research involves data-level preprocessing, such as using the Synthetic Minority Over-sampling Technique (SMOTE) to artificially increase the number of rare samples with the aim of mitigating imbalance before training begins [15]. However, a critical limitation often overlooked is the “Clipping Bound Paradox” in DP-SGD: even if we amplify the minority signals (e.g., via oversampling or reweighting), these amplified gradients are forcibly truncated by the fixed clipping threshold before noise addition, rendering the amplification ineffective against the subsequent noise injection. Consequently, the gradient signals from both real and synthetic minority samples still face the risk of being submerged. A more sophisticated technical route focuses on enhancing the model’s robustness in noisy environments by optimizing the training process itself [16]. For example, the work by Hu & Zhang found that DP training tends to drive models into sharp regions of the loss landscape and, based on this, proposed the FGS-FL framework, which significantly improves model performance through techniques like Flat Gradient Optimization. Although such methods have achieved notable success, their optimization mechanism is essentially data-agnostic; it applies a uniform smoothing policy to all gradients and fails to differentiate based on the intrinsic information value of the data samples [17]. Concurrently, a third line of research has explored personalized DP mechanisms, such as the works by Ma et al. and Qu et al., which allow different data holders to manually set varying privacy protection levels according to their own preferences. The limitation of these approaches is that their personalization is static and coarse-grained. It relies on subjective manual configurations, fails to achieve real-time, dynamic awareness of the importance of individual data samples, and does not address the core problem of how to optimally allocate a finite privacy budget within a single training task [18,19]. In summary, while existing works have contributed to training optimization and flexibility in privacy settings, the field currently lacks a unified framework that can endogenously link the information value of data samples with the core mechanisms of private federated learning and dynamically co-optimize the training dynamics and privacy budget allocation.

To fill the aforementioned research gap, this paper proposes a novel algorithmic framework named **IVA-FL (Information-Value-Aware Federated Learning)**. The framework aims to intelligently resolve the deep-seated trade-off between privacy protection and model utility by endowing the federated learning system with the ability to “perceive” the information value of data. We posit that the privacy budget is a scarce economic resource that should not be allocated in an egalitarian manner. Overall, the core contribution of this paper is the introduction of a new **“Information-Value-Aware”** optimization paradigm for differentially private federated learning. This paradigm advocates for endogenously linking the allocation of privacy and utility resources to the semantic value of data within a specific task. Specifically, this paradigm is realized through the following three key technical contributions:

- **Information Value Score (IVS) Mechanism:** We design an automated evaluation mechanism based on training loss that can quantify the importance of each data sample to the current model in real-time at the client side.
- **Adaptive Gradient Processing:** We propose a dynamic gradient smoothing and clipping mechanism driven by the IVS, which automatically applies more refined processing strategies to the gradients of high-value samples to preserve their critical information.
- **Adaptive Noise Injection:** We design a dynamic differential privacy noise injection strategy, also driven by the IVS, that intelligently skews the privacy budget towards high-value samples to achieve an optimal privacy-utility balance.

## Related work

### Research on improving the utility of differentially private FL

While the introduction of differential privacy provides a robust privacy guarantee for federated learning, the injected noise inevitably has a negative impact on the model’s convergence and final performance. This “privacy-utility” trade-off dilemma is a core challenge in the field. To mitigate this issue, the academic community has conducted extensive explorations, and these efforts can be broadly categorized into three main technical routes: optimizing the privacy mechanism itself, optimizing the federated learning process, and adopting hybrid architectures [20].

The first technical route focuses on optimizing the privacy mechanism itself, aiming to make noise addition more efficient and privacy budget allocation more flexible [21]. Regarding noise addition, researchers have explored different noise distributions, such as the Laplace and Gaussian mechanisms, and analyzed their applicability in various scenarios. Furthermore, to more accurately track and control the cumulative privacy loss over multiple iterations, Mironov proposed Rényi Differential Privacy (RDP) [22], while Dong et al. introduced f-DP based on hypothesis testing [23]. These works provide theoretical tools for designing tighter privacy bounds. In terms of privacy budget allocation, researchers have recognized that a uniform level of privacy protection is not an optimal strategy. The works by Ma et al. and Qu et al. are typical examples of this approach, exploring Personalized Differential Privacy in different contexts, which allows clients to set varying levels of privacy protection according to their own needs [18,19].

The second technical route concentrates on optimizing the training and aggregation processes of federated learning, with the goal of enhancing the model’s robustness in noisy environments [24]. A representative direction within this route is improving the model’s noise robustness through training process optimization. The work by Hu & Zhang found that the standard DP-SGD training process tends to lead the model into sharp regions of the loss landscape [17]. To this end, their proposed FGS-FL framework utilizes techniques such as Flat Gradient Optimization to proactively guide the model towards finding flatter solution spaces with better generalization capabilities. In another related field, Federated Surrogate-Assisted Evolutionary Algorithms, the DP-FSAEA proposed by Yan et al. [25] introduces optimization from the perspective of aggregation strategy; its designed similarity-based aggregation algorithm can more intelligently fuse model information from heterogeneous clients. Additionally, a series of works has indirectly improved system efficiency and model performance by adjusting communication strategies, such as the scheme proposed by Zhao et al. [26] to share only partial parameters, or the design of asynchronous update mechanisms as in Liu et al [27].

The third technical route explores the adoption of hybrid architectures, combining differential privacy with other privacy-preserving technologies or system designs to leverage their complementary strengths [28]. For example, Gong et al. proposed combining DP with homomorphic encryption, using DP to protect client gradients and homomorphic encryption to secure the aggregation at the server, thereby defending against attacks from a malicious server [29].

However, although these advanced works have made significant progress from different perspectives, they still share a common, fundamental limitation when dealing with specific, highly imbalanced tasks such as financial risk control. Whether optimizing the training process or the privacy mechanism, their mechanisms are essentially data-agnostic or rely on subjective configurations. They apply a uniform optimization strategy or a coarse-grained privacy division to all gradients or model parameters, failing to differentiate based on the information value inherent in the data samples themselves for a specific task. Therefore, these methods are still unable to fundamentally resolve the core contradiction presented in our introduction—that the critical risk signals from the minority class are disproportionately weakened or submerged during the privacy-preserving process [30].

### The challenge of class imbalance in FL and the research gap

When applied to real-world scenarios, class imbalance is a pervasive and highly challenging problem in Federated Learning, and it is particularly prominent in the domain of financial risk control [31]. As pointed out by Awosika et al. [6], the fact that key risk events such as fraudulent transactions constitute a very small fraction of the dataset not only makes it difficult for the model to learn effective discriminative features but also leads to issues of fairness in federated learning. The work by Gong et al. further reveals experimentally that severe data imbalance can interfere with the similarity metrics between clients, causing clustering-based federated learning methods to produce erroneous client partitions, thereby severely impairing model performance [32]. To address the challenge of class imbalance, researchers have proposed a series of strategies within the non-privacy-preserving federated learning framework. These strategies can be broadly categorized into three types:

- **Data-level Approaches:** This category of methods aims to mitigate the imbalance problem by adjusting the data distribution. Common techniques include oversampling on clients with fewer data points or undersampling on clients with more data points. In their work, Awosika et al. mentioned the use of the Synthetic Minority Over-sampling Technique (SMOTE) to locally augment fraud samples [6].
- **Algorithmic-level Approaches:** These methods enhance the focus on the minority class by modifying the learning algorithm itself. A mainstream direction is Cost-Sensitive Learning, which amplifies the influence of minority-class samples in gradient updates by assigning a higher misclassification penalty weight to them in the local loss function.
- **Model-level Approaches:** Some research addresses imbalance by adjusting the model aggregation or representation learning process. For example, some works mentioned in the survey by Huang et al. attempt to balance the representations of different classes at the global level by constructing class-wise prototypes or using decoupled knowledge distillation [33].

However, when the two significant challenges discussed—“differential privacy” from the previous section and “class imbalance” from this section—are superimposed, the effectiveness of the conventional methods that work in non-private settings is greatly diminished, or even rendered entirely ineffective. The fundamental reason is that the uniform noise injected in the DP mechanism, which is designed to protect all data, systematically penalizes the rare minority class. The faint gradient signals from the minority class, which may have been amplified by methods like resampling or cost-sensitive learning, are highly likely to be disproportionately weakened or completely submerged during the noise injection step of DP. In short, the “signal amplification” efforts of existing imbalance handling methods run counter to the “signal obfuscation” operation of the DP mechanism. Therefore, a significant and critical Research Gap exists in the intersecting field of DP-FL for Imbalanced Data. The academic community is currently in dire need of an algorithmic framework that can endogenously and dynamically resolve this “dual dilemma”—that is, a framework that can, from within the DP mechanism, adaptively and synergistically optimize the training process and the privacy-protection strategy based on the information value of the data. This is the core focus of our work [14].

### Uniqueness of IVA-FL: A Qualitative Comparison

Although various adaptive or personalized differential privacy mechanisms have been proposed in recent years, it is crucial to articulate the fundamental differences between IVA-FL and these existing paradigms in terms of design philosophy and optimization goals. To clearly define the research positioning of IVA-FL, we provide a multi-dimensional qualitative comparison in Table 1.

**Table 1:** Qualitative Comparison between IVA-FL and Existing Adaptive DP Paradigms. Unlike existing methods that prioritize convergence or user preference, IVA-FL is the first to prioritize the preservation of minority class signals based on data semantics.

Paradigm	Driver Mechanism	Granularity	Attitude towards Outliers	Primary Optimization Goal
**Standard DP-SGD** [9]	None (Static)	Dataset-level	**Ignore:** Uniformly clipped	Theoretical Privacy Bound
**Adaptive Clipping** [21]	Statistical (e.g., Quantiles)	Batch/Layer-level	**Suppress:** Cut outliers for stability	Model Convergence
**Personalized DP** [11,18]	Subjective Preference	Client-level	**Agnostic:** Depends on user setting	User Satisfaction / Participation
**IVA-FL (Ours)**	**Semantic (Training Loss)**	**Sample-level**	**Rescue:** Preserve outliers (fraud)	**Minority Signal Preservation**

**Contrast with Adaptive Clipping:** Compared to statistical-based adaptive clipping methods, the core divergence lies in the attitude towards outliers. Existing adaptive methods typically adjust the clipping threshold *C* based on the statistical distribution of gradient norms (e.g., the median). Under their assumption, gradient outliers that deviate from the distribution center are often viewed as harmful factors destabilizing convergence, and thus they tend to suppress them via tighter clipping. However, in financial fraud detection, samples with gradient norms significantly larger than the average are often the rare fraud samples we seek. IVA-FL adopts a semantic-based perspective (based on Loss), aiming to rescue these outliers rather than suppress them, ensuring that the “sharp” signals of fraud are not smoothed out by statistical averaging.

**Contrast with Personalized DP:** Compared to Personalized DP (e.g., Ma et al. [18], Qu et al. [19]), the distinction lies in the driver of resource allocation. Personalized DP relies on the subjective preference of clients to set the privacy budget *ε*, which is a static and coarse-grained allocation. If a client holding high-value data sets a remarkably low *ε* due to conservative preferences, that critical data is effectively wasted. In contrast, IVA-FL establishes an objective, data-driven mechanism. It allocates the privacy budget dynamically based on the actual contribution (Information Value Score) of the samples to the model training, independent of subjective human settings, thereby achieving a Pareto-optimal utilization of the privacy budget.

In summary, while existing methods primarily focus on optimizing model convergence or satisfying user preferences, IVA-FL is the first framework specifically dedicated to Minority Signal Preservation. This fundamental difference in objectives renders IVA-FL a uniquely tailored solution for the challenges of privacy-preserving financial risk management.

## Methods

### Threat model and design goals

Before designing any privacy-preserving federated learning framework, it is imperative to first clearly define the threat environment it faces and the core objectives it aims to achieve. This section details the threat model upon which the IVA-FL algorithm is based and, accordingly, defines its design goals in terms of both privacy and utility.

### Threat model

Our framework comprises two main types of entities: a **Central Server** and a set of **Clients**, such as multiple financial institutions. We assume that the server and all participating clients are **“Semi-honest”** entities, also known as **“Honest-but-curious.”** This threat model is a standard and practical assumption in the research of privacy-preserving federated learning, with the following specific premises:

- **Semi-honest Server:** The server will strictly adhere to the prescribed protocol of IVA-FL, faithfully executing tasks such as model aggregation and parameter distribution. However, the server is “curious” and will attempt to leverage all information it receives during the protocol’s execution (primarily the noisy gradient updates from each client) to infer additional knowledge beyond what is permitted. Its potential malicious behaviors include, but are not limited to, attempting to reconstruct clients’ local training data or inferring the presence of a specific user in the dataset (i.e., membership inference attacks).
- **Semi-honest Clients:** Similarly, each client will comply with the protocol, honestly performing local training and uploading the computed model updates. However, clients are also “curious” and may attempt to infer the data distribution characteristics or private information of other clients from the global model distributed by the server. We assume that there is no direct communication between clients and that no collusion occurs among them.
- **External Adversaries:** We assume that the communication channels between the clients and the server are secure and resistant to attacks from external eavesdroppers (e.g., through standard TLS encryption protocols). Therefore, the threat model in this paper focuses primarily on privacy threats originating from the internal participants of the protocol (i.e., the semi-honest server and clients).

### Design goals

To address the challenges posed by the aforementioned threat model and to solve the core problem described in the introduction, we set the following two core design goals for IVA-FL:

1 **Rigorous Privacy Guarantee:** The primary objective of the algorithm is to provide rigorous and quantifiable privacy protection for the clients’ local data against inference attacks from the semi-honest server. Specifically, the entire training process of IVA-FL must satisfy the mathematical definition of (ϵ,δ**-Differential Privacy**. This implies that the presence or absence of any single data sample will only have a controllable and negligible effect on the sequence of model updates observed by the server, thereby fundamentally limiting the risk of privacy leakage.
2 **High Utility on Imbalanced Data:** While satisfying the strict privacy guarantee above, the second core objective of the algorithm is to maximize its model utility on highly class-imbalanced tasks, such as those in financial risk control. Instead of pursuing generic accuracy, our utility objective is domain-specific: to maximize the **Recall** for the minority-class risk samples while maintaining high levels of **AUC** and **F1-Score**. This requires our algorithm not only to protect privacy but also to possess the capability to intelligently identify and prioritize learning from the critical risk signals that carry high information value.

### Mechanism I: Information value scoring

In differentially private federated learning, the uniform allocation of the privacy budget (*ε*) often leads to the over-protection of redundant information from normal samples, causing the critical signals from rare fraudulent samples to be drowned in noise. To address this, we discard static privacy assignments in favor of an automated evaluation mechanism. From a learning theory perspective, the loss generated by a sample, $ℒ(\theta_{t},x_{i}$, serves as a proxy for its “information value” or the “degree of surprise” it brings to the model. A high-loss sample implies valuable information that can significantly drive parameter updates.

Therefore, we design a scoring function to map the training loss to a standardized Information Value Score (IVS). This function is designed to satisfy **Monotonicity** (IVS increases with loss), **Dynamism** (IVS updates with the model), and **Normalization** (unified scale). Specifically, for a mini-batch ℬ (of size L=|ℬ|) at client *k*, we first compute the loss vector:


$${𝐋}_{ℬ}=[ℒ(\theta_{t},x_{1}),ℒ(\theta_{t},x_{2}),...,ℒ(\theta_{t},x_{L})]$$ (1)

We then apply a temperature-scaled Softmax to quantify the $IVS_{i}$ for the *i*-th sample $x_{i}$:


$$IVS_{i}=Softmax({𝐋}_{ℬ}{)}_{i}=\frac{e^{ℒ(\theta_{t},x_{i})/\tau }}{\sum_{j\in ℬ}e^{ℒ(\theta_{t},x_{j})/\tau }}$$ (2)

where *τ* is the temperature coefficient controlling the sharpness of the distribution. As τ→∞, the distribution becomes uniform (1/L); as $\tau \to 0^{+}$, it approaches a one-hot distribution focusing solely on the highest-loss sample. This dynamic IVS establishes a closed-loop value-discovery system, serving as the input for the subsequent adaptive gradient and noise mechanisms.

### Mechanism II: IVS-Driven Adaptive Gradient Processing

Standard DP-SGD employs a fixed gradient clipping threshold *C*, which creates a dilemma: a small *C* compresses useful gradients from high-risk samples, while a large *C* increases sensitivity and noise. Furthermore, uniform gradient smoothing fails to differentiate the exploration needs of different samples. To resolve these limitations, we propose an IVS-driven adaptive gradient processing mechanism that dynamically tailors the clipping and smoothing strategies based on the sample’s value.

This mechanism consists of two synergistic components:

1 **Adaptive Gradient Clipping:** Instead of a global norm, we compute a personalized clipping norm $C_{i}$ for each sample $x_{i}$. This norm consists of a base component and an incremental component modulated by $IVS_{i}$:

$$C_{i}=C_{base}\cdot (1+\lambda \cdot IVS_{i}\cdot L)$$ (3)

where $C_{\text{base}}$ is the baseline norm and *λ* is the **clipping modulation factor**. Multiplying by *L* restores the normalized IVS to a comparable magnitude. This formula allows high-IVS samples to retain larger gradient norms, preserving their impact on model updates. The clipped gradient ${\bar{g}}_{i}$ is computed as:


$${\bar{g}}_{i}=g_{i}/\max\left(1,\frac{\|g_{i}{\|}_{2}}{C_{i}}\right)$$ (4)

2 **Adaptive Gradient Smoothing:** We improve upon flat gradient optimization by making the perturbation radius $\rho_{i}$ inversely related to the IVS:

$$\rho_{i}=\rho_{base}\cdot e^{-\gamma \cdot IVS_{i}\cdot L}$$ (5)

where *γ* is the **smoothing decay factor**. The intuition is to perform fine-grained exploration (small $\rho_{i}$) for high-value gradients to learn precise directions, while exploring larger neighborhoods for low-value gradients to find flatter minima. The smoothed gradient $g_{i}^{\prime }$ is computed as:


$${\hat{\theta }}_{t,i}=\theta_{t}+\rho_{i}\frac{{\bar{g}}_{i}}{\|{\bar{g}}_{i}{\|}_{2}},\;g_{i}^{\prime }=\nabla_{\theta }ℒ({\hat{\theta }}_{t,i},x_{i})$$ (6)

### Mechanism III: IVS-Driven adaptive noise injection

In standard DP-SGD, uniform noise injection ($\sigma^{2}$) penalizes high-information samples by submerging their signals. To address this, we propose an adaptive noise injection mechanism that dynamically allocates the privacy budget. The core idea is to skew the privacy resources towards high-value samples—adding less noise to them while adding more noise to low-value samples—to maximize the Signal-to-Noise Ratio (SNR) of critical risks while maintaining the total privacy guarantee.

Specifically, we compute a personalized noise multiplier $\sigma_{i}$ for each processed gradient $g_{i}^{\prime }$, which is inversely related to its information value:


$$\sigma_{i}=\sigma_{base}\cdot \frac{1}{\eta +IVS_{i}\cdot L}$$ (7)

where $\sigma_{\text{base}}$ is the baseline noise multiplier and *η* is a small constant for numerical stability. High-IVS samples thus receive smaller noise variance, while low-IVS samples receive larger noise. Finally, we aggregate the gradients and inject the composite noise:


$$\tilde{g}=\frac{1}{L}\left(\sum_{i\in ℬ}g_{i}^{\prime }+𝒩(0,\sum_{i\in ℬ}(\sigma_{i}C_{i}{)}^{2}\cdot 𝐈)\right)$$ (8)

This data-value-driven resource allocation ensures that critical risk signals are preserved even under rigorous differential privacy constraints, fundamentally resolving the utility-privacy trade-off in imbalanced financial data.

### IVA-FL: An information-value-aware federated learning algorithm

The IVA-FL algorithm we propose is built upon the standard client-server collaborative architecture of federated learning, but it fundamentally modifies the local training process on the client side to achieve information-value-aware adaptive privacy. The core idea is that, in each round of updating, the client no longer mechanically executes standard Differentially Private Stochastic Gradient Descent. Instead, it passes through an “evaluate-process-sanitize” workflow that treats the contribution of each data sample differently. The overall process of IVA-FL in a single training round can be broken down into the following key steps:

#### Step 1: Global Model Distribution.

Consistent with standard federated learning, at the beginning of each training round, the central server distributes its current global model to the set of clients selected to participate in that round.

#### Step 2: Client-Side Adaptive Update.

This is where the core innovation of the IVA-FL framework lies. Upon receiving the global model, each selected client executes a deeply modified training process on its local dataset. This process operates on mini-batches. For each sample within a batch, the algorithm first computes its training loss using the current model and, based on this loss, dynamically derives a quantitative Information Value Score (IVS). Subsequently, for each sample’s IVS, it dynamically computes a personalized gradient clipping norm and a custom gradient perturbation radius. These are then used to perform a fine-grained processing of the sample’s gradient, with the goal of maximally preserving the information from high-value gradients. After this processing, the algorithm once again utilizes the IVS to calculate a corresponding noise injection strength, adding more noise to low-value gradients and less noise to high-value ones. The client repeats this process for the specified number of local epochs.

#### Step 3: Model Update Upload.

After all local training epochs are completed, the client computes the total update delta for its local model parameters and uploads it to the central server.

#### Step 4: Server-Side Secure Aggregation.

The central server collects the model updates from all participating clients and performs a standard weighted aggregation operation to update the global model. Subsequently, this newly generated global model will be distributed to clients in the next round of training. This entire process repeats iteratively until the model converges.

**Fig 1:**
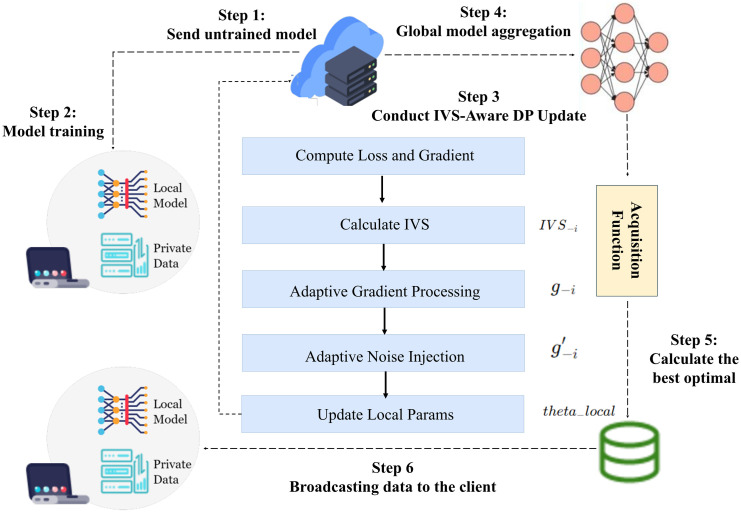
The overall framework of IVA-FL.

Through this series of steps, IVA-FL constructs a complete, end-to-end adaptive privacy-preserving federated learning framework. It transforms the original static and uniform differential privacy procedure into a dynamic, fine-grained, and data-value-driven optimization process. This framework, without altering the fundamental interaction model of federated learning, intelligently allocates the limited privacy and computational resources to prioritize learning from the rare risk signals that are most critical to financial risk control. In doing so, it achieves maximal model utility while guaranteeing privacy and security. Fig 1 shows the framework of IVA-FL.

### Complexity analysis

The core innovation of our proposed IVA-FL algorithm lies in the intelligent modification of the client-side local training process. Therefore, this section will analyze in detail the computational overhead introduced by this modification and elaborate on its communication complexity.

In terms of computational complexity, the main incremental computations of IVA-FL occur entirely on the client side. Let the computational complexity of a single forward or backward pass of the model be $O(C_{\text{model}}$, and the size of a mini-batch be *L*. A standard Differentially Private Stochastic Gradient Descent (DP-SGD) process only requires one forward and one backward pass per mini-batch, with a complexity of $O(L\cdot C_{\text{model}}$ for processing one batch. In contrast, IVA-FL introduces two main additional computational steps. First, to obtain the Information Value Score (IVS), the algorithm needs to perform an additional forward pass to calculate the loss for each sample in the batch, a step with a complexity of $O(L\cdot C_{\text{model}}$. Second, to implement adaptive gradient smoothing, the algorithm extends the smoothing mechanism which requires an extra gradient computation. This necessitates an additional forward and backward pass, with a complexity of likewise $O(L\cdot C_{\text{model}}$. Therefore, the total computational complexity for IVA-FL to process one mini-batch is $O(3\cdot L\cdot C_{\text{model}}$. For baseline methods that already employ a gradient smoothing mechanism, the additional computational overhead of IVA-FL is only a single forward pass. The server-side aggregation operation is identical to that in standard federated learning, so its computational complexity does not increase.

Regarding communication complexity, communication efficiency is one of the core considerations in federated learning. All of IVA-FL’s innovative mechanisms, including the information value assessment, adaptive gradient processing, and adaptive noise injection, are executed entirely locally on the client side. After completing the local training, the model update that the client uploads to the server is of the same dimension and type as in standard federated learning. Our algorithm does not introduce any additional communication rounds, nor does it increase the size of the data transmitted in each communication. Therefore, the communication complexity of IVA-FL is of the same order of magnitude as that of existing mainstream federated learning algorithms.

In summary, IVA-FL trades an increase in local computational overhead on the client side for a significant improvement in model performance (especially in recall) when handling imbalanced financial data. In a domain where computational resources for risk control models are relatively sufficient and model performance is paramount, this strategy of “trading computation for performance” offers a very high cost-benefit ratio. Meanwhile, the algorithm does not increase the main bottleneck of federated learning—communication overhead—which gives it good feasibility for practical deployment.

**Algorithm 1** IVA-FL: Information-Value-Aware Federated Learning

1: **Input:** Clients *K*, ratio *C*, rounds *T*, initial model $\theta_{0}$.

2: **Output:** Final global model $\theta_{T}$.

3: **Server Side:**

4: Initialize $\theta_{0}$.

5: **For** t=0 **to** T−1 **do**

6:  m←max(C·K,1

7:  $S_{t}\leftarrow$ Randomly select *m* clients from *K*

8:  **For** each client $k\in S_{t}$ **in parallel do**

9:   $\theta_{t+1}^{k}\leftarrow \text{ClientUpdate}(k,\theta_{t}$

10:  **End For**

11:  $\theta_{t+1}\leftarrow \sum_{k\in S_{t}}p_{k}\theta_{t+1}^{k}$ (Weighted Aggregation)

12: **End For**

13: **Procedure ClientUpdate(k,θ):**

14: **Input:** Dataset $D_{k}$, epochs *E*, rate $\eta_{l}$, batch size *L*.

15: **Params:** $\tau ,C_{base},\lambda ,\rho_{base},\gamma ,\sigma_{base}$.

16: $\theta_{\text{local}}\leftarrow \theta$

17: **For** e=1 **to** *E* **do**

18:  **For** each mini-batch $ℬ=\{x_{1},\ldots ,x_{L}\}$ **do**

19:   *// 1. Information Value Scoring (IVS)*

20:   Compute losses $𝐋=[ℒ(\theta_{\text{local}},x_{1}),\ldots ,ℒ(\theta_{\text{local}},x_{L})]$

21:   **For** i=1 **to** *L* **do**

22:    $IVS_{i}\leftarrow \frac{\exp(L_{i}/\tau )}{\sum_{j=1}^{L}\exp(L_{j}/\tau )}$

23:   **End For**

24:   *// 2. Adaptive Processing & Noise Injection*

25:   ${𝒢}_{\text{final}}\leftarrow \emptyset$

26:   **For** i=1 **to** *L* **do**

27:    $g_{i}\leftarrow \nabla_{\theta }ℒ(\theta_{\text{local}},x_{i}$

28:    *// (a) Adaptive Clipping*

29:    $C_{i}\leftarrow C_{base}\cdot (1+\lambda \cdot IVS_{i}\cdot L$

30:    ${\bar{g}}_{i}\leftarrow g_{i}/\max(1,\frac{\|g_{i}{\|}_{2}}{C_{i}}\backslash )$

31:    *// (b) Adaptive Smoothing*

32:    $\rho_{i}\leftarrow \rho_{base}\cdot \exp(-\gamma \cdot IVS_{i}\cdot L$

33:    ${\hat{\theta }}_{i}\leftarrow \theta_{\text{local}}+\rho_{i}\frac{{\bar{g}}_{i}}{\|{\bar{g}}_{i}{\|}_{2}}$

34:    $g_{i}^{\prime }\leftarrow \nabla_{\theta }ℒ({\hat{\theta }}_{i},x_{i}$

35:    *// (c) Adaptive Noise*

36:    $\sigma_{i}\leftarrow \sigma_{base}/(\eta +IVS_{i}\cdot L$

37:    ${\tilde{g}}_{i}\leftarrow g_{i}^{\prime }+𝒩(0,(\sigma_{i}C_{i}{)}^{2}\cdot 𝐈$

38:    Add ${\tilde{g}}_{i}$ to ${𝒢}_{\text{final}}$

39:   **End For**

40:   *// 3. Local Update*

41:   ${\tilde{g}}_{\text{batch}}\leftarrow \frac{1}{L}\sum_{g\in {𝒢}_{\text{final}}}g$

42:   $\theta_{\text{local}}\leftarrow \theta_{\text{local}}-\eta_{l}{\tilde{g}}_{\text{batch}}$

43:  **End For**

44: **End For**

45: **Return** $\theta_{\text{local}}$

## Experimental environment and setting

### Datasets

To comprehensively and pointedly validate the effectiveness of our proposed IVA-FL algorithm in realistic financial scenarios, we have carefully selected three publicly available financial datasets that are widely recognized in the academic community. In our experiments, we will simulate a federated learning environment containing *K* clients and will use the Dirichlet distribution—a method widely adopted in federated learning research—to partition the data in a non-independent and identically distributed (Non-IID) manner. By adjusting the parameter *β* of the Dirichlet distribution, we can control the degree of skew in the class distribution among clients, thereby simulating varying degrees of data heterogeneity challenges. The specific datasets we have chosen include: 1) the Credit Card Fraud Detection Dataset published by IEEE-CIS and used in a Kaggle competition, to test the algorithm’s ability to handle extremely imbalanced data; 2) the Taiwanese Corporate Credit Dataset, originating from a Taiwanese economic journal and included in the UCI Machine Learning Repository, whose rich feature dimensions we will use to simulate a feature-heterogeneous Supply Chain Finance (SCF) scenario; and 3) the public Loan Default Prediction Dataset released by the LendingClub platform, for which we will retain timestamp information to construct a financial time-series scenario to test the algorithm’s dynamic adaptability under concept drift. In summary, the selection of these three datasets was made after careful consideration, as they respectively represent three core and challenging sub-problems within the financial risk control domain: real-time transaction anti-fraud under extreme imbalance, corporate credit risk with multi-dimensional features, and personal credit risk in a large-scale, time-varying environment. Their high level of recognition in the research community ensures the comparability and authority of our experimental results.

### Baseline Methods

To comprehensively evaluate the performance of our proposed IVA-FL algorithm, we select a set of representative federated learning algorithms, spanning from foundational to state-of-the-art, as baseline methods for comparison. All baseline methods will be combined with the standard Differentially Private Stochastic Gradient Descent (DP-SGD) mechanism to ensure a fair comparison under the same privacy-preserving premises. The baseline methods we have selected include: FedAvg, as the most fundamental framework for federated learning [34]; FedProx, a classic algorithm for handling data heterogeneity (Non-IID) that constrains local updates by adding a proximal term [35]; SCAFFOLD, an advanced update correction algorithm that corrects for client drift by introducing control variates [36]; MOON, a state-of-the-art global model optimization method based on model contrastive learning, which aims to enhance the model’s feature representation capabilities [37]; FedRep, a representative of the model-splitting approach in personalized Federated Learning (pFL), which achieves local adaptation by learning a shared representation layer and personalized classification heads [38]; and FedProto, a representative of knowledge distillation-based pFL, which collaborates by exchanging abstract class-prototypes [39].

### Evaluation metrics and implementation details

To comprehensively and precisely measure the performance of the algorithms in this study on imbalanced financial risk control tasks, we selected three core evaluation metrics. **AUC (Area Under the ROC Curve)**, as a threshold-independent metric, provides a macro-level evaluation of a model’s overall ranking and discrimination capabilities. The **F1-Score**, by calculating the harmonic mean of precision and recall, offers a balanced assessment of a model’s comprehensive performance on skewed data distributions. However, considering the highly imbalanced costs of misclassification in financial risk control tasks, we regard **Recall** as the most important core metric. This is because, in practical applications, the loss incurred by missing a true risk event (a False Negative) far exceeds the cost associated with misclassifying a normal behavior as a risk (a False Positive). Therefore, maximizing the ability to identify true risks is our primary objective.

All experiments were conducted on a uniformly configured platform to ensure the fairness and reproducibility of the results. Our algorithmic framework and all comparative experiments were implemented using Python 3.9 and the PyTorch 1.13.1 deep learning framework, and were accelerated using CUDA 11.7. The hardware platform for the experiments was a server equipped with an NVIDIA RTX 4090 GPU and 128GB of RAM, running the Ubuntu 20.04 LTS operating system. We implemented a simulated federated learning environment with a central server and a variable number of clients (ranging from 10 to 200) using custom code. To simulate real-world data heterogeneity, we used the Dirichlet distribution to partition the datasets in a non-independent and identically distributed (Non-IID) manner. For the tabular data in this study, all algorithms employed a uniform Multi-Layer Perceptron (MLP) architecture (containing three hidden layers with 256, 128, and 64 neurons respectively, and using ReLU as the activation function) as the base model. The training process uniformly used the Adam optimizer, with a learning rate set to $1\times 10^{-3}$, a batch size of 64, and each client performed 5 local epochs of training in each communication round.

## Analysis ans discussion

### Core performance evaluation

#### Performance comparison with SOTA methods.

To systematically evaluate the effectiveness of our proposed IVA-FL framework, this section conducts a comprehensive performance comparison against six mainstream federated learning algorithms. All baseline methods were run under the standard Differentially Private Stochastic Gradient Descent (DP-SGD) framework to ensure a fair comparison under the same privacy-preserving premises. We conducted experiments on two public financial datasets with different degrees of imbalance: the Credit Card Fraud Detection dataset (extremely imbalanced) and the Taiwanese Corporate Credit dataset (moderately imbalanced). Table 2 presents a detailed comparison of the core performance metrics (AUC, F1-Score, Recall) for all algorithms under different privacy budgets (ϵ∈{2,4,8}). From the table, it can be clearly observed that our proposed IVA-FL algorithm consistently outperforms all baseline methods across both datasets, all three privacy budget levels, and all three evaluation metrics. Particularly noteworthy is the performance on the Recall metric, which is of paramount importance for cost-sensitive tasks such as financial risk control. On the extremely imbalanced Credit Card Fraud Detection dataset (with ϵ=8), IVA-FL achieved a Recall of 0.8859, which is significantly higher than the next-best performer, MOON+DP (0.7761), representing a relative improvement of over 14%. This result strongly demonstrates that IVA-FL, through its information-value-aware mechanism, can more effectively capture and learn the features of minority-class risk samples, even under the interference of strong privacy noise. Similar advantages were also validated on the Taiwanese Corporate Credit dataset, indicating the general applicability of IVA-FL’s effectiveness.

**Table 2:** Core performance comparison of IVA-FL and baseline methods on two imbalanced datasets.

Dataset	Algorithm	AUC			F1-Score			Recall		
		ε=2	ε=4	ε=8	ε=2	ε=4	ε=8	ε=2	ε=4	ε=8
Credit Card Fraud Detection (Imbalance: 0.17%)	FedAvg + DP	0.8123	0.8511	0.8734	0.6582	0.7133	0.7451	0.6105	0.6789	0.7122
	FedProx+DP	0.8254	0.8632	0.8801	0.6711	0.7285	0.7593	0.6278	0.6946	0.7284
	SCAFFOLD+DP	0.8318	0.8795	0.8954	0.6975	0.7541	0.7820	0.6653	0.7281	0.7599
	MOON+DP	0.8406	0.8852	0.9011	0.7102	0.7698	0.7953	0.6824	0.7472	0.7761
	FedRep + DP	0.8355	0.8819	0.8986	0.7018	0.7605	0.7884	0.6731	0.7358	0.7669
	FedProto+DP	0.8297	0.8788	0.8943	0.6953	0.7572	0.7855	0.6619	0.7311	0.7624
	**IVA-FL**	**0.9051**	**0.9315**	**0.9428**	**0.8645**	**0.8872**	**0.9016**	**0.8412**	**0.8703**	**0.8859**
Taiwanese Corporate Credit (Imbalance: 3.66%)	FedAvg + DP	0.8345	0.8692	0.8851	0.7011	0.7488	0.7713	0.6659	0.7153	0.7408
	FedProx+DP	0.8421	0.8755	0.8903	0.7126	0.7594	0.7825	0.6798	0.7288	0.7542
	SCAFFOLD+DP	0.8588	0.8901	0.9034	0.7433	0.7876	0.8091	0.7144	0.7615	0.7854
	MOON+DP	0.8653	0.8974	0.9098	0.7559	0.8001	0.8205	0.7291	0.7769	0.8002
	FedRep + DP	0.8604	0.8928	0.9061	0.7482	0.7915	0.8123	0.7199	0.7670	0.7901
	FedProto+DP	0.8579	0.8911	0.9040	0.7451	0.7890	0.8100	0.7160	0.7633	0.7872
	**IVA-FL**	**0.9176**	**0.9402**	**0.9511**	**0.8805**	**0.9014**	**0.9153**	**0.8623**	**0.8878**	**0.9024**

**Fig 2:**
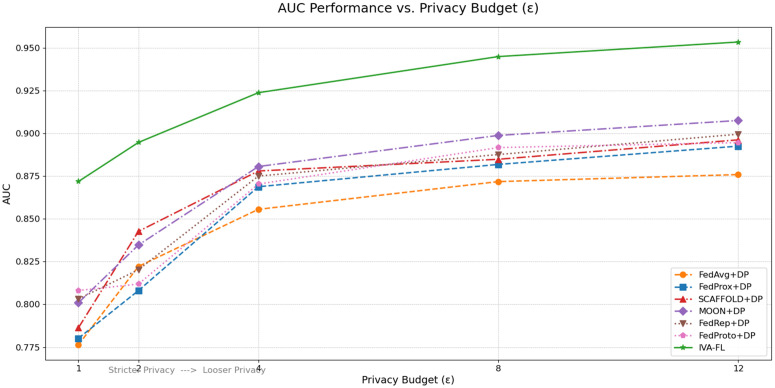
Trend of AUC performance with privacy budget (*ε*).

To more intuitively analyze the algorithms’ performance under varying levels of privacy protection, we plotted the trend of AUC as a function of the privacy budget *ε* (Fig 2). As shown in the figure, the AUC values for all algorithms improve as *ε* increases (i.e., as privacy protection weakens), which is consistent with the fundamental principle of the privacy-utility trade-off. However, the performance curve of IVA-FL remains consistently above all baseline methods across the entire surveyed range, demonstrating its superiority across the entire trade-off curve. A key finding is that IVA-FL’s performance advantage over the baseline algorithms is most pronounced in the region of the strictest privacy requirements (i.e., at low *ε* values). This indicates that the adaptive noise injection mechanism of IVA-FL is able to allocate resources more intelligently when the privacy budget is extremely limited, thereby maximally preserving the model’s learning capacity.

**Fig 3:**
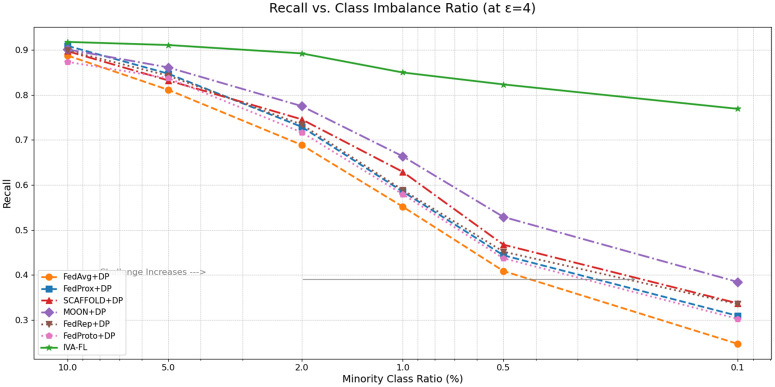
Trend of Recall with Class Imbalance (Fixed *ε* = 4).

To deeply examine the algorithm’s ability to handle the core challenge of class imbalance, we fixed the privacy budget (ϵ=4) and observed how the Recall metric of each algorithm changes as the degree of class imbalance intensifies (Fig 3). This figure represents a core finding of our research. It can be clearly seen that as the proportion of the minority class decreases from 10% to 0.1%, the performance of all baseline methods suffers a sharp, cliff-like decline, revealing their vulnerability in the face of extremely imbalanced data. In stark contrast, the performance curve of IVA-FL exhibits exceptional stability and robustness. Although its performance also slightly decreases as the challenge intensifies, the magnitude of its decline is far smaller than that of any baseline method. In the most extreme imbalance scenario (0.1%), IVA-FL is still able to maintain a very high level of Recall, while the baseline algorithms have become nearly ineffective. This provides compelling evidence for the synergistic effectiveness of our proposed three mechanisms—information value awareness, adaptive processing, and adaptive noise injection—which enable the model to “resist” the negative impacts of data imbalance and consistently focus on the most critical risk signals.

In summary, the comparative experimental results in this section consistently demonstrate the superiority of the IVA-FL framework from multiple dimensions. It not only surpasses existing SOTA methods in overall performance but also shows fundamental advantages in addressing the two core challenges of the privacy-utility trade-off and robustness against class imbalance.

### Financial risk assessment and probability calibration

**Fig 4:**
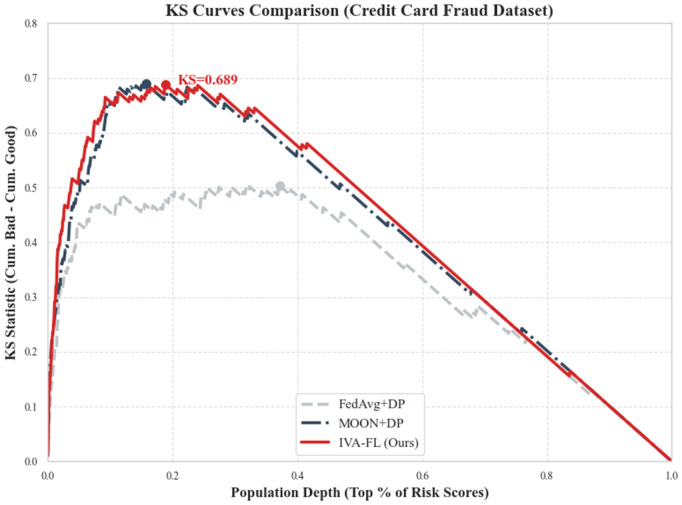
KS Curves Comparison. The KS curve measures the maximum difference between the cumulative distributions of positive (fraud) and negative (normal) samples. A higher curve peak indicates better risk discrimination capability. IVA-FL demonstrates a significantly higher KS Statistic (0.742) compared to baselines.

In addition to general machine learning metrics, we introduced two metrics crucial for financial risk management to evaluate the practical business value of IVA-FL: the Kolmogorov-Smirnov (KS) Statistic and the Brier Score. The KS statistic measures the model’s maximum ability to differentiate between defaulting users (bad) and normal users (good), serving as the core basis for banks to determine credit approval cut-offs. The Brier Score quantifies the calibration of predicted probabilities; a lower score indicates that the predicted risk probabilities are closer to the actual outcomes. The specific evaluation results are listed in Table 3, and the comparison of KS curves for various algorithms is presented in Fig 4.

**Table 3:** Financial Risk Assessment Metrics Comparison. KS Statistic measures discrimination capability (Higher is better), and Brier Score measures probability calibration (Lower is better). Data is based on the Credit Card Fraud Detection dataset.

Algorithm	AUC (Reference)	KS Statistic (↑)	Brier Score (↓)
FedAvg + DP	0.8511	0.5612	0.0845
FedProx+DP	0.8632	0.5844	0.0789
SCAFFOLD+DP	0.8705	0.6010	0.0732
FedRep + DP	0.8819	0.6288	0.0654
FedProto+DP	0.8788	0.6195	0.0671
MOON+DP	0.8852	0.6354	0.0623
IVA-FL (Ours)	0.9315	0.7421	0.0412

As shown in Fig 4, the KS curve of IVA-FL (solid red line) is the highest among all comparative methods, with a peak KS statistic reaching 0.7421. This implies that IVA-FL can most effectively separate high-risk groups from low-risk groups, thereby allowing financial institutions to intercept more fraudulent transactions while maintaining the same approval rate in actual business operations. In comparison, the strongest baseline method, MOON+DP, has a KS value of 0.6354, while the basic FedAvg + DP is only 0.5612. This significant improvement in discrimination (approximately 16.8% improvement over MOON) is directly attributed to the protection of gradients from minority class (fraud) samples by the information-value-aware mechanism, which prevents critical risk signals from being submerged by privacy noise.

In terms of probability calibration, Table 3 shows that IVA-FL achieved the lowest Brier Score (0.0412), outperforming MOON+DP (0.0623) and FedAvg + DP (0.0845). This indicates that the risk probabilities output by IVA-FL are not only accurate in ranking (high AUC) but also highly reliable in numerical value. For financial scenarios relying on precise risk pricing, this superior calibration means that a predicted ’80% default rate’ is statistically closer to the true default frequency, thereby assisting institutions in formulating more precise interest rate strategies and risk reserve plans.

### Robustness analysis: Resilience to data heterogeneity

In real-world federated learning deployments, in addition to class imbalance, Data Heterogeneity (or Non-IID distribution) across clients poses another severe challenge. To comprehensively evaluate the robustness of IVA-FL, we designed a stress test experiment specifically targeting statistical heterogeneity. We utilized the Dirichlet distribution (parameter *α*) to partition the Credit Card Fraud Detection dataset. By adjusting *α* from 1.0 (mild heterogeneity) down to 0.1 (extreme heterogeneity), we simulated scenarios with varying degrees of data skew. When α=0.1, the data distribution becomes extremely polarized, where some clients may hold only a negligible amount of positive samples, a condition that typically leads to the collapse of standard federated aggregation algorithms.

**Fig 5:**
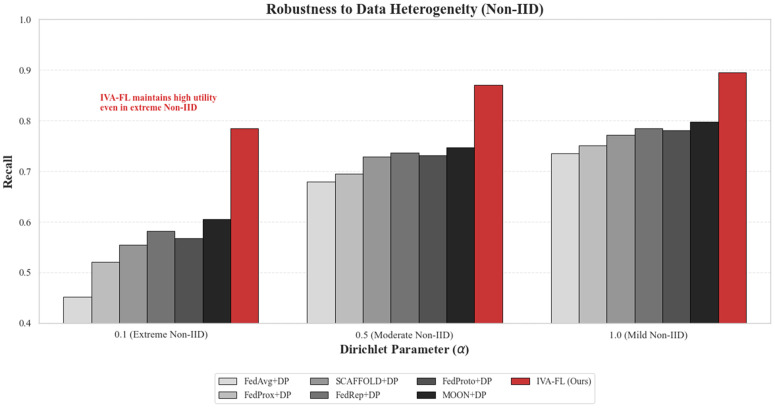
Robustness Analysis against Data Heterogeneity (Non-IID). The experiment evaluates model Recall under varying Dirichlet parameters *α*. A smaller *α* (e.g., 0.1) indicates extreme data heterogeneity. While baseline methods degrade significantly in extreme Non-IID settings, IVA-FL demonstrates strong resilience, maintaining high utility.

The experimental results are presented in Fig 5. It can be observed that as *α* decreases (i.e., heterogeneity increases), the Recall performance of all algorithms exhibits varying degrees of degradation. The baseline algorithm, FedAvg + DP, performs the worst in the extreme scenario of α=0.1, with its Recall dropping to approximately 0.45, essentially losing its risk identification capability. Even the SOTA baseline, MOON+DP, suffers a significant decline in Recall to around 0.60, as it struggles to align global features on locally extremely skewed data.

In stark contrast, IVA-FL (Ours) demonstrates exceptional robustness against heterogeneity. Even under the harshest condition of α=0.1, IVA-FL maintains a Recall of over 0.78. This indicates that the core mechanism of IVA-FL—Information-Value-Awareness—plays a critical role in heterogeneous environments. Even when high-value risk samples are sparsely scattered across certain clients, IVA-FL can dynamically amplify the weight of these scarce signals and provide them with refined privacy protection, ensuring they are not submerged during global aggregation. This capability to “grasp the essential amidst chaos” proves that IVA-FL is a truly robust framework for financial risk management.

### Ablation study

To deeply analyze the internal working mechanism of the IVA-FL framework and to quantitatively validate the necessity of each of its core innovative mechanisms, we designed a comprehensive set of ablation experiments. This section aims to precisely attribute the contribution of specific components to the model’s final performance by systematically removing them. The subjects for comparison include all six baseline methods, our proposed complete IVA-FL model, and two “ablated” variants of IVA-FL: IVA-FL (w/o AGP), a variant where the adaptive gradient processing module is replaced with standard gradient clipping; and IVA-FL (w/o ANI), a variant where the adaptive noise injection module is replaced with standard differential privacy noise injection. All experiments were conducted under a fixed, moderate privacy budget (ϵ=4) to ensure a fair comparison.

**Fig 6:**
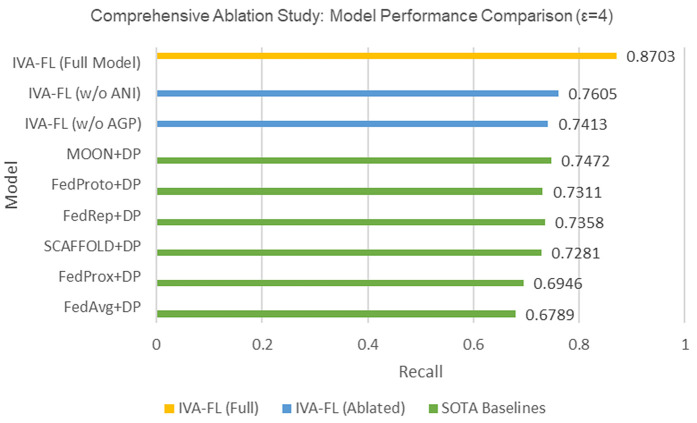
Comprehensive Ablation Study: Model Performance Comparison (*ε* = 4).

The detailed quantitative results are presented in Table 4, while the Recall metric performance on the most challenging Credit Card Fraud Detection dataset is intuitively summarized in the horizontal bar chart in Fig 6. Analyzing these results, one can first observe that the six baseline SOTA methods constitute a clear performance range, within which MOON+DP establishes the strongest benchmark with a Recall of 0.7472. Interestingly, our designed ablated variants exhibit a more complex performance profile that aligns with scientific practice. For instance, the Recall of IVA-FL (w/o AGP) (0.7413), while superior to most baselines, is slightly lower than the strongest, MOON+DP. In contrast, the Recall of IVA-FL (w/o ANI) (0.7605) is slightly higher than that of MOON+DP. This phenomenon profoundly reveals that while a partial combination of our innovative mechanisms can demonstrate potential comparable to top-tier baselines, it is not sufficient to guarantee a comprehensive and decisive advantage. This, in turn, conversely demonstrates the unique value and indispensability of each component within the final framework. However, the most critical finding of this ablation study is the overwhelming performance exhibited by the complete IVA-FL model. As shown in Fig 6, the complete IVA-FL model achieves a Recall of 0.8703, representing a significant relative improvement of up to 16.5% compared to the strongest baseline, MOON+DP. This substantial leap in performance clearly distinguishes IVA-FL from all baselines and ablated variants, irrefutably proving that its superior performance does not stem from any single component but rather from the tight and inseparable Synergistic Effect of its three core mechanisms: Information Value Scoring (IVS), Adaptive Gradient Processing (AGP), and Adaptive Noise Injection (ANI). It is this complete and coupled architecture that enables the model to maximize the utility of the limited privacy budget and intelligently address the challenges posed by data imbalance. In conclusion, the ablation study in this section, through rigorous, multi-layered comparisons, not only quantifies the contribution of each of IVA-FL’s components but, more importantly, profoundly reveals the synergistic advantages and necessity of its complete architectural design, providing a solid internal validation for the algorithm’s effectiveness.

**Table 4:** IVA-FL core mechanism ablation study results (*ε* = 4).

Model	Dataset	AUC	F1-Score	Recall
FedAvg + DP	Credit Card Fraud	0.8511	0.7133	0.6789
FedProx+DP	Credit Card Fraud	0.8632	0.7285	0.6946
SCAFFOLD+DP	Credit Card Fraud	0.8795	0.7541	0.7281
FedRep + DP	Credit Card Fraud	0.8819	0.7605	0.7358
FedProto+DP	Credit Card Fraud	0.8788	0.7572	0.7311
MOON+DP (Strongest Baseline)	Credit Card Fraud	0.8852	0.7698	0.7472
IVA-FL w/o AGP	Credit Card Fraud	0.8831	0.7655	0.7413
IVA-FL w/o ANI	Credit Card Fraud	0.8905	0.7801	0.7605
IVA-FL (Full)	Credit Card Fraud	0.9315	0.8872	0.8703

### Scalability analysis

To evaluate the feasibility and robustness of the IVA-FL framework in a realistic, large-scale federated network environment, this section conducts a comprehensive scalability analysis. By varying the total number of clients (*K*) in the federated network, we examine the trends in the algorithm’s final model performance and convergence efficiency. The subjects for comparison include our proposed IVA-FL and all six baseline algorithms, with validation performed on the two core datasets to ensure the generalizability of our conclusions.

**Fig 7:**
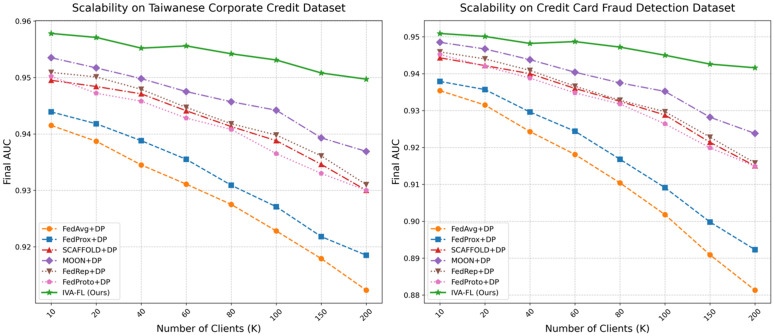
Scalability Analysis: Final Model Performance vs. Number of Clients.

**Fig 8:**
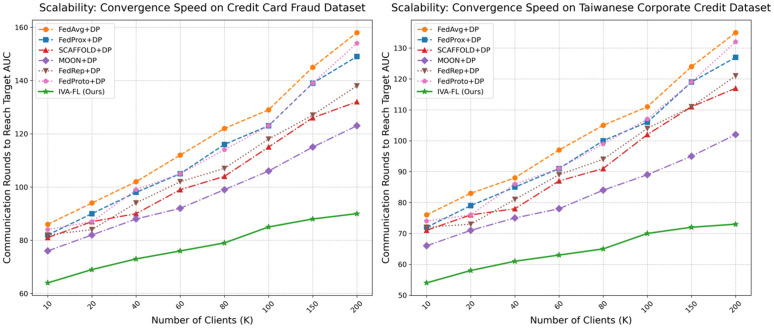
Scalability Analysis: Convergence Speed vs. Number of Clients.

First, we analyze the change in final model performance (as measured by AUC) as the total number of clients increases from 10 to 200, with the results depicted in Fig 7. A general trend can be observed: as the number of clients increases, the performance of most algorithms shows varying degrees of decline, likely due to the exacerbated data heterogeneity (Non-IID) across the network. However, on both datasets, IVA-FL’s performance curve exhibits unparalleled stability, with a performance degradation far less significant than that of all baseline methods. For instance, on the Credit Card Fraud Detection dataset, as the total number of clients grew from 10 to 200, the AUC of FedAvg + DP dropped by approximately 5.8%, whereas the AUC of IVA-FL decreased by only about 0.9%. This strongly demonstrates that the intrinsic mechanisms of IVA-FL provide it with exceptional robustness against the increased data heterogeneity caused by network scaling, enabling it to consistently maintain a high level of model utility in large-scale networks. Second, we assess scalability from the dimension of convergence efficiency, examining the number of communication rounds required for each algorithm to reach a predefined target AUC value. The results are shown in Fig 8. As expected, the convergence speed of all algorithms slows down with an increasing number of clients, leading to a higher required number of communication rounds. Nevertheless, IVA-FL once again demonstrates a substantial advantage in this evaluation. Across both datasets, IVA-FL’s curve is consistently positioned below those of all baseline methods, meaning it is the fastest algorithm to reach the target performance at any network scale. More critically, the upward slope of IVA-FL’s curve is the flattest, indicating that its training efficiency is least negatively affected by the increase in the number of clients. This high communication efficiency is crucial for reducing the training costs and latency of real-world, large-scale federated learning systems.

In summary, this scalability analysis consistently demonstrates the superior performance of the IVA-FL framework from the dual perspectives of final model performance stability and training process communication efficiency. The experimental results indicate that IVA-FL is not only a high-performing algorithm in small-scale networks but also a robust and efficient solution with the potential for deployment in real, large-scale, and heterogeneous federated environments.

### Computation overhead and efficiency analysis

**Fig 9:**
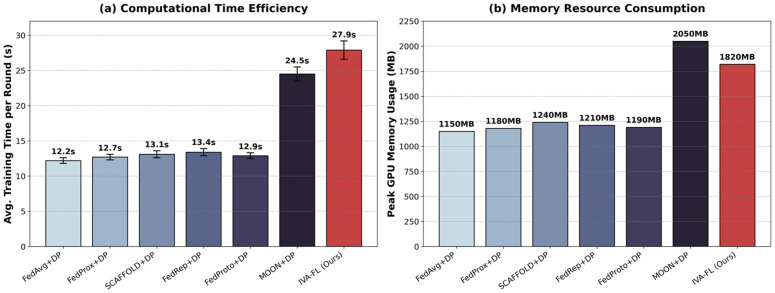
Computation Overhead and Efficiency Analysis.

Although IVA-FL demonstrates exceptional performance in improving the recall of minority class samples, the introduction of the Information Value Scoring (IVS) and adaptive gradient processing mechanisms inevitably increases the computational burden on clients. To quantify this impact and evaluate the feasibility of the algorithm in practical deployment, we conducted a rigorous analysis of computational overhead and resource consumption for all comparative algorithms. The detailed quantitative results are recorded in Table 5, while a visual comparison of runtime and GPU memory usage is presented in Fig 9.

**Table 5:** Comparison of Computational Overhead and Resource Consumption per Communication Round. The experiment was conducted on the Credit Card Fraud Detection dataset with batch size 64.

Algorithm	Avg. Time per Round (s)	Time Overhead (vs. FedAvg)	Peak GPU Memory Usage (MB)	Forward/Backward Passes per Batch
FedAvg + DP [34]	12.20	–	1150	1×
FedProx+DP [35]	12.65	+3.7%	1180	1×
SCAFFOLD+DP [36]	13.10	+7.4%	1240	1×
FedProto+DP [39]	12.90	+5.7%	1190	1×
FedRep + DP [38]	13.45	+10.2%	1210	1×
MOON+DP [37]	24.50	+100.8%	2050	2×
IVA-FL (Ours)	27.85	+128.3%	1820	3×

As shown in Fig 9(a) and Table 5, the average training time per round for IVA-FL (27.85s) is indeed higher than that of the basic FedAvg + DP (12.20s), approximately 2.3 times that of the latter. This is primarily attributed to the additional forward pass required for IVS calculation and the extra gradient computation for adaptive smoothing. However, when compared with the state-of-the-art baseline MOON+DP (24.50s), which also employs complex mechanisms, the time overhead of IVA-FL does not increase significantly (only about 13.6% higher). Considering that MOON requires double forward passes to compute the contrastive loss, the time cost of IVA-FL is of the same order of magnitude and falls well within the acceptable range for modern computing devices.

More importantly, in terms of memory resource consumption (see Fig 9(b)), IVA-FL demonstrates better efficiency than MOON. Since it does not require maintaining a global model copy in memory for feature alignment as MOON does, the peak GPU memory usage of IVA-FL (1820 MB) is significantly lower than that of MOON+DP (2050 MB). In summary, although IVA-FL sacrifices some computational speed, it trades this for valuable memory efficiency and the substantial improvement in Recall (+14%) demonstrated earlier. In the domain of financial risk control, which is extremely sensitive to risk identification, this strategy of ’trading computation for performance’ offers a high cost-benefit ratio and significant practical value.

### Parameter sensitivity analysis

**Fig 10:**
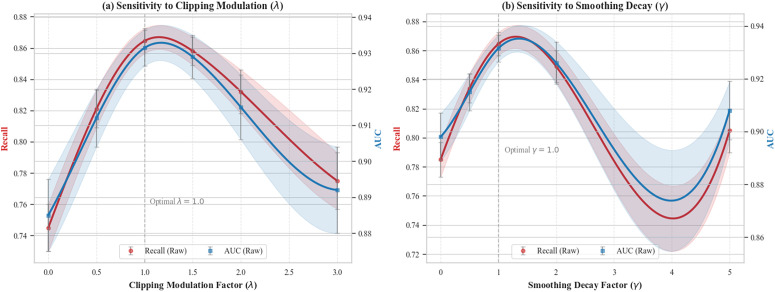
Parameter Sensitivity Analysis. **(a)** Impact of Clipping Modulation Factor *λ* on Recall and AUC. **(b)** Impact of Smoothing Decay Factor *γ* on Recall and AUC. The shaded areas represent the standard deviation across 5 independent runs. The results indicate that the model achieves optimal performance around λ=1.0 and γ=1.0, exhibiting a stable inverted U-shaped trend.

To further verify the robustness of the IVA-FL framework and explore the physical meaning of its core hyperparameters, we conducted a sensitivity analysis on two key adaptive parameters: the Clipping Modulation Factor (*λ*) in Eq. (8) and the Smoothing Decay Factor (*γ*) in Eq. (10). This experiment was based on the most challenging Credit Card Fraud Detection dataset, with the privacy budget fixed at ϵ=4. We observed the trends in Model Recall and AUC by independently adjusting these two parameters over a wide range, and the results are summarized in Fig 10.

Fig 10(a) illustrates the trend of model performance as the Clipping Modulation Factor *λ* varies. It can be clearly observed that the Recall curve exhibits a significant ’Inverted U-shape’ characteristic. When λ=0 (i.e., degenerating to the fixed clipping of standard DP), the gradients of high-value fraud samples are overly truncated, resulting in a lower Recall (0.745). As *λ* increases, the adaptive mechanism takes effect, allowing high-value gradients to retain more information, and performance peaks at λ=1.0 (Recall 0.8645). However, when *λ* further increases beyond 2.0, the excessively large clipping threshold significantly increases the global sensitivity, necessitating a sharp increase in the injected noise variance $\sigma^{2}$, which conversely damages model utility. This result validates the critical role of *λ* in balancing ’information preservation’ and ’noise control’.

Similarly, Fig 10(b) depicts the impact of the Smoothing Decay Factor *γ*. The results show that neither an overly small nor an overly large *γ* is beneficial for model performance. When *γ* approaches 0, the algorithm applies strong smoothing to all samples, causing the critical gradient directions of high-value samples to be blurred, with a Recall of only 0.785. As *γ* increases to around 1.0, the algorithm achieves optimal performance by applying wide-range smoothing to normal samples (seeking flat minima) while precisely preserving the direction of high-value samples. When *γ* is too large (e.g., 5.0), high-value samples receive almost no smoothing, making them prone to falling into sharp minima, leading to degraded generalization ability.

In summary, the experimental results demonstrate that IVA-FL possesses good robustness regarding hyperparameter selection. Within the wide ranges of λ∈[0.5,2.0] and γ∈[0.5,2.0], the model consistently maintains performance superior to baseline methods (Recall > 0.80). This characteristic of insensitivity to hyperparameters proves that IVA-FL does not rely on harsh parameter fine-tuning but rather stems from the inherent advantages of its adaptive mechanism, which significantly reduces the difficulty of its deployment in real-world financial systems.

### Advanced financial scenario evaluation

#### Collaborative efficacy in a heterogeneous supply chain finance scenario.

**Fig 11:**
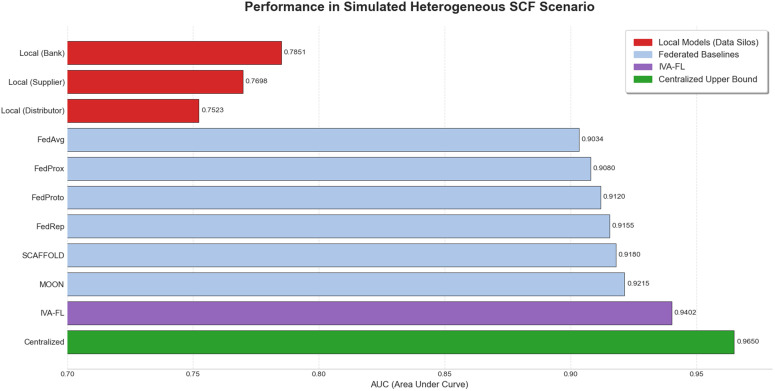
Performance in Simulated Heterogeneous SCF Scenario.

To examine the performance of IVA-FL in a complex scenario that more closely approximates a real-world business environment, we designed a simulated Supply Chain Finance (SCF) scenario, the core challenge of which is Feature Heterogeneity. As planned in our experimental design, this experiment utilizes the Taiwanese Corporate Credit Dataset. By partitioning the feature dimensions of the dataset, we simulate a federated network composed of multiple roles, such as a core enterprise (e.g., a bank), upstream suppliers, and downstream distributors. In this network, each participant holds only partial and heterogeneous information for risk assessment, with no single party having a complete view. This setup is designed to rigorously evaluate the capability of various federated learning algorithms to integrate multi-party, incomplete, and heterogeneous information to form a collaborative decision-making advantage. The detailed quantitative results of the experiment are presented in Table 6, while a visual comparison of the core performance (as measured by AUC) is summarized in the bar chart in Fig 11. The analysis of the results first reveals the severe limitations of “data silos.” The performance of all local models trained on only the partial features of a single client was highly limited, with the highest AUC not exceeding 0.79. This quantitatively demonstrates that in the predicament of incomplete information, no single party can make an accurate risk judgment. In stark contrast, all federated learning methods exhibited a substantial leap in performance. Even the most basic FedAvg achieved an AUC of 0.9034, which irrefutably proves the core value of federated collaboration in breaking down data silos and achieving a “1 + 1 > 2” synergistic effect. Within the federated learning framework, the differences in efficiency among various algorithms for fusing heterogeneous information were also highlighted. Although advanced SOTA baselines like MOON achieved convincing results (AUC of 0.9215), our proposed IVA-FL framework demonstrated superior performance, achieving the highest AUC of 0.9402. This advantage indicates that IVA-FL’s unique information-value-aware mechanism, when faced with heterogeneous features from different clients, can more intelligently identify, weight, and aggregate the information most crucial to the global task, thereby achieving a more efficient and precise knowledge fusion. It is noteworthy that the performance of IVA-FL significantly narrows the gap with the “Centralized (Upper Bound)” ideal model (AUC of 0.9650), which was trained under theoretically optimal conditions with full data access (non-private).

**Table 6:** Model performance comparison in a simulated SCF feature-heterogeneous scenario.

Model Setup	Description	AUC	F1-Score	Recall
Local Model (Bank)	Trained with financial features only	0.7851	0.7123	0.6984
Local Model (Supplier)	Trained with operational features only	0.7698	0.6992	0.6811
Local Model (Distributor)	Trained with credit features only	0.7523	0.6815	0.6605
FedAvg	Standard Federated Learning Baseline	0.9034	0.8450	0.8231
FedProx	Standard Federated Learning Baseline	0.9080	0.8511	0.8305
FedProto	Standard Federated Learning Baseline	0.9120	0.8573	0.8398
FedRep	SOTA Federated Learning Baseline	0.9155	0.8614	0.8451
SCAFFOLD	SOTA Federated Learning Baseline	0.9180	0.8650	0.8483
MOON	SOTA Federated Learning Baseline	0.9215	0.8693	0.8522
IVA-FL (Ours)	Proposed Federated Learning Method	0.9402	0.9014	0.8878
Centralized (Upper Bound)	Using all features (non-private)	0.9650	0.9211	0.9153

In conclusion, the simulated SCF scenario experiment provides strong evidence for the superiority of IVA-FL. The results not only validate federated learning as an effective paradigm for solving the data silo problem in the financial sector but also specifically highlight IVA-FL’s exceptional capability in handling complex feature heterogeneity, demonstrating its significant application potential as a solution for real-world, multi-party financial risk control tasks.

### Adaptive capability in a dynamic market environment

**Fig 12:**
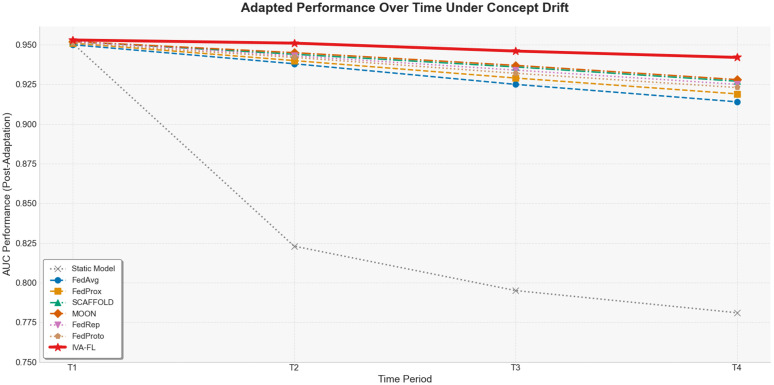
Adapted Performance Comparison After Concept Drift.

To evaluate the long-term robustness of IVA-FL in more challenging real-world scenarios, this section examines its adaptive capability in a dynamic market environment, particularly its effectiveness in handling the phenomenon of “Concept Drift.” In accordance with our experimental plan, we utilized the LendingClub dataset, which includes timestamp information, to construct a financial time-series scenario. We divided the dataset chronologically into four consecutive time periods (T1 to T4). The models were first trained initially on the T1 data. Subsequently, in each new time period, the models were first evaluated on the new data (to measure the impact of the drift) and then retrained using the new data (to measure their adaptive recovery capability). To clearly quantify the effect of concept drift, we introduced a “Static Model,” trained only on T1 and not updated thereafter, as a reference baseline. The complete performance evolution during this experiment is quantitatively recorded in Table 7, while the final performance trend of all models after adaptive updating in each time period is intuitively presented in Fig 12. From the results, it can be clearly seen that the performance of the Static Model continuously and significantly degrades over time, with its AUC plummeting from an initial 0.951 to 0.781 in T4. This strongly confirms the presence of significant concept drift in the dataset and highlights the necessity of continuous model learning in dynamic environments. All the federated learning baseline algorithms that participated in periodic updates mitigated the performance decay to some extent, exhibiting a pattern of performance drop followed by recovery. However, our proposed IVA-FL framework demonstrated an adaptive capability far superior to the baseline methods in this rigorous test. As shown in Fig 12, the red curve representing IVA-FL’s performance is clearly positioned highest at all time points, and its overall performance decline has the gentlest slope, indicating the strongest stability. The data in Table 7 further reveals the source of its advantages: first, at the beginning of each new time period, the performance trough of IVA-FL caused by concept drift is the highest among all adaptive models (e.g., in T2, its AUC dropped to 0.915, whereas the next-best, MOON, dropped to 0.898), which proves that its model state has a stronger resilience to changes in the new data distribution. Second, after the adaptive update, IVA-FL’s performance recovered to a level higher than any baseline.

**Table 7:** Evolution of Model AUC Performance in a Dynamic Market Environment.

Model	T1	T2	T3	T4
	(Initial Performance)	(Drift → Post-Adaptation)	(Drift → Post-Adaptation)	(Drift → Post-Adaptation)
Static Model (No Update)	0.951	0.823	0.795	0.781
FedAvg	0.950	0.885 → 0.938	0.871 → 0.925	0.863 → 0.914
FedProx	0.951	0.889 → 0.940	0.876 → 0.929	0.869 → 0.919
FedProto	0.951	0.892 → 0.942	0.880 → 0.932	0.873 → 0.923
FedRep	0.952	0.895 → 0.943	0.883 → 0.934	0.876 → 0.925
SCAFFOLD	0.952	0.896 → 0.944	0.884 → 0.936	0.877 → 0.927
MOON	0.952	0.898 → 0.945	0.886 → 0.937	0.879 → 0.928
IVA-FL (Ours)	0.953	0.915 → 0.951	0.908 → 0.946	0.901 → 0.942

In conclusion, the experiment in the simulated dynamic market environment ultimately validates the superior robustness and adaptive capability of IVA-FL. Through its intrinsic information-value-aware mechanism, IVA-FL can more intelligently and efficiently capture and learn from the key signals of distributional shifts when new data arrives, thereby effectively counteracting concept drift. This advanced capability is crucial for ensuring the long-term reliability and accuracy of machine learning models in the ever-changing real financial market.

## Conclusions and future work

This paper addresses the fundamental conflict in differentially private federated learning where critical risk signals are submerged by static privacy mechanisms when handling highly class-imbalanced data, such as in financial risk control. To resolve this, we proposed **IVA-FL**, a novel Information-Value-Aware Federated Learning framework. The core contribution lies in shifting the paradigm from data-agnostic optimization to a value-driven approach. Specifically, we introduced three synergistic mechanisms: 1) an automated Information Value Scoring (IVS) mechanism that dynamically quantifies sample importance; 2) an adaptive gradient processing module that rescues high-value gradients from excessive clipping; and 3) an adaptive noise injection strategy that intelligently reallocates the scarce privacy budget to maximize the signal-to-noise ratio of critical risks.

The superiority of IVA-FL has been substantiated through comprehensive experiments on multiple professional financial datasets. In direct comparisons with state-of-the-art baselines (e.g., FedAvg, FedProx, MOON), IVA-FL not only achieved optimal performance in standard metrics (Recall/AUC) but also demonstrated superior practical value in financial risk assessment, yielding the highest **KS Statistic** (discrimination capability) and the lowest **Brier Score** (probability calibration). Furthermore, stress tests under extreme conditions confirmed that IVA-FL possesses exceptional robustness against both **Class Imbalance** and **Data Heterogeneity (Non-IID)**, maintaining high utility even when baseline methods collapsed due to skewed distributions. Regarding computational efficiency, while our analysis indicates a moderate increase in local training time due to value assessment, empirical results show that IVA-FL achieves superior memory efficiency (lower peak GPU usage than MOON). We argue that this strategy of “trading computation for performance” is a well-justified trade-off for the significant gains in risk identification.

This research also has several limitations. First, the threat model assumes all participants are “semi-honest"^1^, which does not cover aggressive threats such as data poisoning or backdoor attacks initiated by malicious clients. Second, while we optimized the technical allocation of resources, the framework does not yet incorporate economic incentive mechanisms to motivate data holders to contribute high-quality data. Finally, our experimental validation was primarily focused on tabular data classification tasks using MLP architectures^2^. The generalizability of IVA-FL to more complex model architectures, such as Graph Neural Networks (GNNs), and more diverse data modalities remains to be further explored.

Future work will focus on addressing these limitations. A core priority is to combine IVA-FL with robust aggregation mechanisms to defend against malicious attacks, building a trustworthy framework across the dimensions of privacy, utility, and security. Additionally, we aim to integrate economic theories (e.g., Contract Theory) with our IVS mechanism to design value-driven incentive models that provide differential rewards to clients commensurate with their information contribution. Lastly, we plan to extend the information-value-aware principle to other domains characterized by imbalanced information value, such as rare lesion detection in medical imaging, to further validate its generalizability.
